# Informed consent instead of assent is appropriate in children from the age of twelve: Policy implications of new findings on children’s competence to consent to clinical research

**DOI:** 10.1186/s12910-015-0067-z

**Published:** 2015-11-09

**Authors:** Irma M. Hein, Martine C. De Vries, Pieter W. Troost, Gerben Meynen, Johannes B. Van Goudoever, Ramón J. L. Lindauer

**Affiliations:** Department of Child and Adolescent Psychiatry, Academic Medical Center, Meibergdreef 5, 1105 AZ Amsterdam, Netherlands; Department of Medical Ethics and Health Law, Leiden University Medical Center, PO Box 9600, 2300 RC Leiden, Netherlands; Faculty of Philosophy, VU University Amsterdam, De Boelelaan 1105, 1081 HV Amsterdam, Netherlands; Tilburg Law School, Tilburg University, Prof. Cobbenhagenlaan 221, 5037 DE Tilburg, Netherlands; Academic Medical Center, Emma’s Children Hospital, Meibergdreef 9, 1105 AZ Amsterdam, Netherlands; Department of Pediatrics, VU University Medical Center, De Boelelaan 1105, 1081 HV Amsterdam, Netherlands

**Keywords:** Minors, Mental competence, Informed consent, Assessment, Health care policy

## Abstract

**Background:**

For many decades, the debate on children’s competence to give informed consent in medical settings concentrated on ethical and legal aspects, with little empirical underpinnings. Recently, data from empirical research became available to advance the discussion. It was shown that children’s competence to consent to clinical research could be accurately assessed by the modified MacArthur Competence Assessment Tool for Clinical Research. Age limits for children to be deemed competent to decide on research participation have been studied: generally children of 11.2 years and above were decision-making competent, while children of 9.6 years and younger were not. Age was pointed out to be the key determining factor in children’s competence. In this article we reflect on policy implications of these findings, considering legal, ethical, developmental and clinical perspectives.

**Discussion:**

Although assessment of children’s competence has a normative character, ethics, law and clinical practice can benefit from research data. The findings may help to do justice to the capacities children possess and challenges they may face when deciding about treatment and research options. We discuss advantages and drawbacks of standardized competence assessment in children on a case-by-case basis compared to application of a fixed age limit, and conclude that a selective implementation of case-by-case competence assessment in specific populations is preferable. We recommend the implementation of age limits based on empirical evidence. Furthermore, we elaborate on a suitable model for informed consent involving children and parents that would do justice to developmental aspects of children and the specific characteristics of the parent-child dyad.

**Summary:**

Previous research outcomes showed that children’s medical decision-making capacities could be operationalized into a standardized assessment instrument. Recommendations for policies include a dual consent procedure, including both child as well as parents, for children from the age of 12 until they reach majority. For children between 10 and 12 years of age, and in case of children older than 12 years in special research populations of mentally compromised patients, we suggest a case-by-case assessment of children’s competence to consent. Since such a dual consent procedure is fundamentally different from a procedure of parental permission and child assent, and would imply a considerable shift regarding some current legislations, practical implications are elaborated.

## Background

In clinical practice an accurate assessment of children’s decision-making capacities is needed to avoid two pitfalls: to impose complex medical decisions on children who are unable to make them, and to inadvertently exclude capable children who want to take part in decision-making [1]. For many decades, the debate on children’s competence to give informed consent or assent in medical settings concentrated around ethical and legal aspects, with little empirical underpinnings [2]. Generally speaking, the term decision-making capacity is used to describe different levels of patients’ abilities, and the term competence refers to the degree of capacity that is sufficient to allow patients to make an autonomous medical decision [3]. We will apply this terminology in this article. Furthermore, when we indicate competence to consent, we also consider competence to refuse or dissent. Child assent refers to affirmative agreement of a minor who is to take part in the informed consent procedure in a way adapted to his or her capabilities, while their legal representative has the formal role of consenting [4].

In clinical practice many questions remained unanswered, for example which age span to evaluate, how to study the full range of abilities relevant to children’s decision-making described in the literature, how to assess decision-making capacities regarding different types of medical decisions, and how to objectively assess children’s competence. Progress was hard to achieve in debates on the subject and the lack of consensus on children’s competence to consent was reflected by the restricted clinical implementation of the concept. There was a gap between recommendations regarding policies for children’s involvement in the consent procedure and what had been documented in scientific research about children’s competence assessment. The empirical approach emerged as a designated way to examine the dilemmas.

Recently, objective data stemming from empirical research on children’s competence to consent became available, offering an opportunity to further the discussion. An earlier study conducted by the authors demonstrated in a sample of 161 pediatric patients that children’s decision-making capacities regarding clinical research could be assessed in a valid and reliable way by means of an instrument, the modified MacArthur Competence Assessment Tool for Clinical Research (MacCAT-CR) [5]. The MacCAT-CR is a semistructured interview format developed by Appelbaum and Grisso in 2001, which measures the four aspects of decision-making capacities that reflect the standards for competence in most jurisdictions (understanding the disclosed information about the nature and procedures of the research; reasoning in the process of deciding about participation; appreciation of the effects of research participation on the patient’s own situation; and expressing a choice about participation) [6]. In the same study, the four domains representing competence in most jurisdictions (understanding, appreciation, reasoning and expressing a choice) appeared to constitute a single trait or continuum of competence in children, which allowed for estimating a cutoff score on MacCAT-CR above which competence was likely. This is in contrast with adult literature, stating that scores on subscales need to be weighted independently, and that failure in one domain could translate into an incompetent assessment [3]. In adults, because of this presumption, dimensionality was never tested.

Age limits for children to be deemed competent to decide on research participation were estimated: children of 11.2 years and above generally appeared to be competent, while children of 9.6 years and younger were not. Children between 9.6 and 11.2 years were in a transition period; they develop important capacities but their maturity is not pervasive [5]. Furthermore age turned out to be the factor that explains most of the variance in children’s competence to consent, followed by intelligence. Theoretical assumptions that risk and complexity of the decision would be related to a competence classification could not be confirmed with empirical data [7]. This demonstrated that more radical decisions, requiring a higher level of competence, could possibly be made by children as young as the group of children who were able to make lower impact decisions. An explanation might be that children at a certain age have the required capacities, and competent decision-making is possible when information provision is of good quality. For other potential determining factors for competence, like gender, systemic influences, disease experience, ethnicity and socio-economic status, no clear relationship with a competence classification could be demonstrated either. Interestingly, parents appeared to judge their child more readily competent than experts would [7].

Obviously, research on competence to consent to research in children must be extended, and the next indicated step is to test if the model produced from this initial research can be replicated in a testing dataset. Bearing this in mind, we will start to consider the meaning of these recent empirical findings in view of their context. Since the age limits for asking children’s consent stated in many jurisdictions do not coincide with those demonstrated in our research [5], we need to evaluate whether it would be advisable to reset local statutory age-limits. Having the possibility to assess children’s competence individually in a standardized way, an alternative option (namely to let go of rigid age limits for alleged competence and switch to a case-by-case assessment) might be considered. For example, now that it is possible to establish a very intelligent eight-year old boy’s decision-making capacities, we need to consider if it would be judicious to do so and if decision-making competent, to allow him to give informed consent. Although the assessment instrument proved to be accurate, there might be possible drawbacks of the normative classification of children into groups of competent and incompetent ones. Overall, we should evaluate whether the clinical assessment of children’s competence by an instrument is comprehensive, or that we miss out on important non-measurable factors. Finally, we need to consider if we are fully aware of the influence of developmental aspects affecting children’s competence, and if this makes children’s competence different from adults.

In this article we will reflect on possible implications of the recent empirical findings on children’s competence to consent considering normative, developmental, and clinical perspectives. Subsequently, we will derive recommendations for policies.

## Discussion

### Normative aspects

Considering children either competent or incompetent is a normative judgment. However, the fact that competence is a normative judgment does not mean that it cannot be informed by research data. Research shows that a competence assessment can be reliably performed using a structured tool like the MacCAT-CR. The MacCAT-CR’s total and sub-scores showed a good reproducibility and the overall accuracy of MacCAT-CR scores in correctly classifying children as competent against the reference standard was high as well [5]. In addition, it was shown that using such a tool, three age groups could be distinguished: one in which children are most probably incompetent, one in which children are most probably competent, and a group in which probability of (in) competence is less clear (between 9.6 and 11.2 years). Such findings do not prescribe how ethics and law should deal with (in) competence and children. But, as we will discuss below, the findings may help to do justice to the capacities children possess and challenges they may face when deciding about treatment and research options. For instance, for health care professionals, as well as parents, it is important to know that a structured and reliable tool for assessing competence in children is available. Performing such a structured competence assessment may clarify the capacities of an individual child in case professionals have doubt about the child’s competence. In addition, the findings concerning the age groups may support the development of guidelines dealing with informed consent in children. Still, clearly, the ethical and legal norm for competence in children cannot be directly derived from these research findings. For instance, establishment of cutoff scores for competence is after all based on normative judgments.

### Ethical aspects

#### Rational reasons *versus* emotions and values

Some authors have raised doubts about the validity of competence assessment by MacCAT-scales, and argued that the MacCAT-assessment puts the main emphasis on rational reasoning. Ethicists and other commentators bring into the discussion the role of values and emotions in competence. Hope and colleagues [8] suggest that to develop a better understanding of competence, research needs to be expanded by factors of competences not covered by the four criteria that are commonly applied (understanding, appreciation, reasoning, expressing a choice). Charland argues that MacCAT-scales seldom sufficiently recognize emotive components and values in decision-making competence [9]. He states that “pathological values” may be present in patients with specific psychiatric disorders that effect competence. He proposes to incorporate a measure of emotional competence into a competence assessment instrument before considering it a valid measure. Appelbaum, author of MacCAT-T, agrees that emotions aid humans in processing information but suggests that the feasibility of adding emotional capacity to the list of capacities essential for decisional competence should be demonstrated first [10]. No consensus in this debate has been reached yet.

Furthermore, children may differ from adults by not having developed yet stable long term goals and values in life, meaning that children may procedurally be classified as competent although their decisions are based on values that might change. This could imply that later on they might regret decisions based on those early-life values. It is conceivable that in children “immature values” might be present that are not covered by competence assessment using MacCAT-scales. The study on accuracy of MacCAT-CR in children was performed using a reference standard established by experts. In cases of psychiatric disorders the pathological values might be recognized by clinical experts, in children we might expect the clinical experts to have recognized immature values when present in children. If not, the study might have missed out on an unmeasured component of children’s competence. This would then have resulted in considering more children competent using the MacCAT-CR than actually justified.

### Legal aspects

#### Age-limits *versus* case-by-case assessment

It is widely recognized that the evolving abilities of children and adolescents are reflected by a gradual development of decision-making capacities [2]. The use of a fixed age-limit as cutoff for competence is defendable, knowing that age is an efficient indicator of competence with considerable practical advantages as an administrative and normative gauge. It can be measured easily and offers a clear framework. However, the disadvantage of fixed age-limits is the all or nothing character, meaning that relevant differences between individuals are not taken into account. With a set age-limit, some incompetent individuals above the limit will unjustly be deemed competent and some competent individuals below the limit unjustly deemed incompetent.

An alternative for the fixed age-limit is a case-by-case assessment of decision-making competence. A recent study has shown that doctors and researchers tend to judge a child to be competent if the child’s decision conforms to their own ideas of the child’s best interest [11]. This means that competence is gauged by the outcome of the decision rather than by the process of reasoning in deciding about participation. Data suggest that unstructured performance of competence assessments is often sub-optimal and hence the reliability of unstructured judgments has been poor [12]. To avoid this bias, a case-by-case assessment would require an objective assessment instead of the currently used intuitive one. The MacCAT-CR would be an appropriate instrument for this purpose in the research context [5].

#### Reset age-limits

Age-limits for asking children’s consent vary widely over nations and states [5]. In Europe, domestic law determines whether or not people are competent to consent to healthcare interventions [13]. In some countries autonomous decision-making is lawful only from 18 years onwards and in other countries minors are allowed to take healthcare decisions from a fixed age below legal majority, e.g., 12 years in the Netherlands and 15 years in Denmark [13]. Another variant applied in most Canadian provinces and Switzerland is a flexible system stating that anyone who is capable can give informed consent, whereby competence is evaluated on a case-by-case basis [13]. In the United States, generally speaking, it often falls to parents or legal guardians to provide informed permission for medical decisions and children under 18 are to give assent [14]. Ideally, age-limits accomplish the goal of striking a proper balance in order to both protect children's interests when they are not fully able to do so themselves and to respect their autonomy when they can exercise it. So if a fixed age-limit is used, it must be generally in accordance with the developmental stages. Earlier studies conducted by the authors now offer scientific input for setting a reasonable and just age-limit; as far as we currently know the age-limit that presents closest accordance with children’s competence is eleven or twelve years.

#### Best interest

Clearly, the duty to protect the best interests of the child (see UN Convention on the Rights of the Child, Art 3) is also relevant in this context. Although we will not go into the (legal) details of this article, we would like to offer the following line of reasoning: If a child possesses all the required decision-making capacities which means that it understands the relevant information, is able to appreciate the consequences of the decision, capable of reasoning and of expressing a choice, in other words if a child is considered competent to give informed consent, that would mean that a child is capable of acting in its best interest. Children who are not decision-making competent yet, should not be burdened with the responsibility for decisions they are not able to make autonomously. Others should then decide in their best interests.

### Developmental aspects

#### Difference between competence assessment in adults and children

In adults, patients are deemed competent unless the clinician has reasons to believe otherwise. In children, it is generally the other way around, they are presumed not to be competent in most jurisdictions [14]. Whereas in adults MacCAT-scales are merely used to ascertain incompetence in mentally compromised patients out of an overall competent population, in children it might be more important to recognize competent patients in a mainly incompetent population. The application of MacCAT-scales in children puts higher demands on the specificity of the instrument; it serves to weed out the proportion of children that are correctly identified as competent from those (possibly incorrectly) identified as incompetent. In the MacCAT-CR study, specificity in children of 11.2 years and older was good: 90 % [5].

#### Parent *versus* professional

Research showed that judgments of incompetence by parents frequently coincided with the MacCAT-CR incompetent classification, however parents’ assessments of competence showed only moderate agreement with the MacCAT-CR standard. This might imply that parents express a higher expectation regarding their children’s competence, assigning them more voice and responsibility, than professionals do. In literature the opposite was described: in a sample of 120 young people undergoing orthopedic surgery in 1993, health professionals recommended a much lower mean age for competence than parents did (10.3 *vs*. 13.9) [15]. The recent finding that parents judged their children more readily competent than clinicians, might be related to the specific dynamics of parent-child relationships [16]. Good parents are expected to inhibit their child’s impulsive, risky, and sometimes harmful behavior. They substitute the child’s ineptitude and inability to judge situations with their superior judgment. Parents tailor their parenting behavior to the specific abilities of the child. Children who are raised in a warm and understanding atmosphere are often able to present their part in a joint decision-making process at an early stage of their development [17]. An authoritative parenting style, which includes direction-giving and limit-setting, is positively related with an adolescent’s capacity for autonomous decision-making [18]. In the medical context children might be capable of autonomous decision-making, albeit, within the guiding environment set by their parents. Possibly parents assign their children more decision-making competence than professionals do, because parents shape the family context and professionals regard the child more independently.

#### Assessment must cover developmental aspects

Differences between children and adults regarding decision-making competence have been found in the ability to restrain impulsivity and in the ability to place a given decision in a larger temporal context [19]. The inadequate capacity of children in risk assessment could be connected to the late full maturation of the frontal lobes that are essential for effective executive functions [18]. In addition, research shows that adolescents generally do not fully possess the capacity to appreciate the long-term consequences of their choices until the age of 21 [18]. Research demonstrated a difference between decision-making under low levels of arousal or in situations with low emotional upheaval (cold cognition), and thought processes under high levels of arousal and emotional valance (hot cognition) [20]. Hot cognition may result in intuitive responses rather than carefully considered, rational responses [20]. Decisions on clinical research participation involving information provision, rehearsal of information, time to consider, and reflection with parents, generally result in cold cognition decisions. Treatment decisions are more prone to hot cognition when involving time pressure or weighty risks. With the research results showing that children of 11.2 years and above have comparable decision-making capacities to adults concerning research participation, clearly normative aspects play a role in the assessment of when their decision-making competence was good enough. The developmental progress as described above, is expected to further improve children’s decision-making competence with age, so at 11.2 years it is supposedly not as good as it can get. Furthermore, we need to consider children’s possible immaturity in decisions of a supervisory or managerial nature normally made by their parents, for example overseeing the family agenda, or arranging transport to the hospital. Possibly, children are able to decide with cold cognition on research participation, but are less able to responsibly respond to, for example, unforeseen traffic situations or stressful peer-interactions. Therefore they still need the dyadic relationship with parents who provide the necessary direction-giving and limit-setting.

### Recommendations and practical aspects

Considering the before-mentioned new empirical findings on children’s competence to consent, the normative, ethical, developmental, and clinical perspectives, we will now envisage some recommendations which we deduced. From a practical point of view, assessment of all pediatric patients’ competence on a case-by-case basis with an instrument would impose a heavy burden on patients, professionals, and the medical system. A selective implementation of a standardized competence assessment in exceptional cases would be preferable over a broad implementation.

For the research context, under the age of 9.6 years children were generally incompetent to decide on research participation [5], so an individual assessment does not seem profitable. Children between 9.6 and 11.2 years were in the change-over period, an individual assessment of competence might be applicable in this age group. Children of 11.2 years and above can generally be considered decision-making competent, and although they need a supportive context, no individual assessment is needed. In special research populations where there are reasons to doubt children’s decision-making capacities (e.g. intellectual disabled children or pediatric patients with a psychiatric disorder that diminishes competence), a research protocol could include a standardized competence assessment of participants. In these cases an assessment could prevent incompetent patients from the unjustified burden of decision-making responsibility.

In the treatment context, there are no conclusive age-limits for competence established empirically. The MacCAT-T is an instrument for assessing patient’s competence to consent in a treatment setting, measuring the same four aspects of decision-making capacities as the MacCAT-CR. In a pilot study, use of the MacCAT-T proved feasible in a population of children between 8 and 17 years of age who had to decide on predictive genetic testing for cardiac diseases [21]. Although sample-size was small (N = 17) and conclusions premature, all participating children above the age of 12 years were judged to be competent to decide on this treatment option. We suggest that it may be valuable to create the possibility for clinicians to take into account exceptional cases, such as the assessment of a child under the age of 12, seemingly competent, who has to make a weighty decision. In such cases an individual standardized competence assessment could underpin the exception to the rule.

Parents are generally provided with the legal authority to raise their children, assigning them rights and responsibilities. In some circumstances a legal representative or guardian will carry this role as opposed to parents, in this article we will include them as we speak of parents. To achieve a balanced consideration between the legal position of the child and that of the parents, a dual consent procedure (child and parent) is recommended for minors from the age of 12 until the age they are allocated rights for independent consent. Even if we establish a child’s decision-making competence regarding the medical decision at hand, a dual consent procedure will do justice to developmental aspects of children and the specific characteristics of the parent-child dyad. The parental role is needed to offer extra protection by creating the context for the child’s competent decision-making and by facilitating the child’s long term autonomy.

Besides the advantages of a dual consent procedure, there may be a disadvantage concerning possible disagreement between child and parent, which may require elaborated policies. In the Dutch situation experience has been gained with a dual consent procedure and evaluation shows that disagreement between parent and child was not a concern [22, 23]. In case of disagreement, all efforts must be made to reach agreement. In some exceptional cases of disagreement, Dutch law allows for carrying through the decision of a competent child above the age of 12 when it can prevent serious harm. This could also be the case if restraining from diagnostic testing would imply a loss of important treatment possibilities.

Practically, a dual consent procedure would imply that the patient information form would need to consist of two separate versions, one for the parents and one for the child, each followed by an informed consent form to be signed.

A dual consent procedure is fundamentally different from a procedure of parental permission and child assent, and would imply a considerable shift regarding some current legislations, for instance within the EU context [2]. Likewise, in the current Code for Federal regulations of the United States [4] by definition children are “persons who have not attained the legal age for consent to treatments or procedures involved in the research” (45CRF46.402 (a)). The legal age of adulthood is a matter of local law, but is in a large majority of states 18 years. Regulations state that some children might be able to give their assent, meaning an affirmative agreement. However, in research the institutional review board may still waive the assent requirement under certain circumstances (45 CFR 46.116). Some authors have proposed that children’s assent should only be required from a fixed age of 14 years, based on theories of subject autonomy and child development [24]. The empirical evidence that children are generally competent not only to assent, but even to give consent from the age of 12 contradicts these regulations and theories.

There is no indication of a considerable difference in children’s development between regions with widely varying policies regarding children’s consent. These local variations in regulations may have evolved under the influence of historical, cultural, or emotional preferences, representing a local normative view. Empirical data now provide underpinnings for more evidence-based age limits in policies.

### Limitations and directions for future research

Although our previous empirical research provides substantial data to consider in debate and practice, many aspects of children’s decision-making competence are still to be studied, of which we will name just a few. For instance, regarding medical decision-making, the age limits for reaching legal majority vary between countries and states from 16 to 21 years. Research does not show at what age a dual consent procedure will no longer prove effective. In addition, more research is needed to demonstrate the validity of a cutoff score on a standardized assessment instrument for competence and the desirability of such a cutoff must be considered. In the treatment setting, more extended research on reliability and validity of the MacCAT-T in children is recommended. The importance of children’s decision-making competence is not confined to the medical context alone but may be of significance to adjacent fields, for instance children’s competence to proceed to criminal adjudication or to be consulted in civil procedures, which requires further research. Furthermore, new developments in neuropsychiatry may contribute to the understanding of the functioning of specific brain regions or connections that promote competent decision-making.

### Summary

Research outcomes show that the legal concept of medical decision-making competence could be operationalized into a standardized assessment instrument for children in the clinical context. The MacCAT-CR proved accurate for children’s competence assessment in clinical research. Developmental aspects, especially the fine-tuning of decision-making within the parent-child dyad, including the broader family context, are of importance in addition to a standardized competence assessment.

Policy recommendations include a selective implementation of individual assessment of children’s competence in medical decision-making by a standardized tool in combination with practicable, generally appropriate age-limits. In the research context children can be deemed competent from the age of 12 and above, and a case-by-case assessment of competence might be valuable in children in the change-over period between 10 and 12, and in case of children older than 12 years when there are reasons to doubt their competence for instance because of mental disabilities. In the treatment context, individual competence assessment might create an opportunity in exceptional cases to allow a competent child under the age of 12 to co-decide over significant medical interventions. A dual consent procedure, including both child as well as parents, is recommended for children from the age of 12 until they reach majority.

## Abbreviations

MacCAT-CR: MacArthur Competence Assessment Tool for Clinical Research
MacCAT-T: MacArthur Competence Assessment Tool for Treatment
